# Docetaxel and Doxorubicin Codelivery by Nanocarriers for Synergistic Treatment of Prostate Cancer

**DOI:** 10.3389/fphar.2019.01436

**Published:** 2019-12-18

**Authors:** Ke Li, Wenhua Zhan, Yulong Chen, Rajiv Kumar Jha, Xueli Chen

**Affiliations:** ^1^ Shaanxi Key Laboratory of Brain Disorders, Shaanxi Key Laboratory of Ischemic Cardiovascular Disease, Institute of Basic and Translational Medicine, Xi’an Medical University, Xi’an, China; ^2^ Department of Radiotherapy, General Hospital of Ningxia Medical University, Yinchuan, China; ^3^ College of Clinical Medicine, Xi’an Medical University, Xi’an, China; ^4^ Engineering Research Center of Molecular and Neuro Imaging of the Ministry of Education, School of Life Science and Technology, Xidian University, Xi’an, China

**Keywords:** prostate cancer, synergistic chemotherapy, codelivery nanoparticles, docetaxel, doxorubicin

## Abstract

Combination chemotherapy has been proven to be an efficient strategy for the treatment of prostate cancer (PCA). However, the pharmacokinetic distinction between the relevant drugs is an insurmountable barrier to the realization of their synergistic use against cancer. To overcome the disadvantages of combination chemotherapy in the treatment of PCA, targeted nanoparticles (NPs), which can codeliver docetaxel (DOC) and doxorubicin (DOX) at optimal synergistic proportions, have been designed. In this study, the DOC and DOX codelivery nanoparticles (DDC NPs) were constructed by hyaluronic acid (HA) and cationic amphipathic starch (CSaSt) through a self-assembly process. Human PCA cell lines (PC-3, DU-145, and LNCap) and mouse models were then used for evaluation *in vitro* and *in vivo*, respectively, of delivery and antitumor effects. The DDC NPs were spherical with rough surfaces, and the size and zeta potential were 68.4 ± 7.1 nm and -22.8 ± 2.2 mV, respectively. The encapsulation efficiencies of DOC and DOX in the NPs were 96.1 ± 2.3% and 91.4 ± 3.7%, respectively, while the total drug loading was 9.1 ± 1.7%. Moreover, the ratio of DOC to DOX in the DDC NPs was approximately 1:400, which aligned with the optimal synergistic proportions of the drugs. The DDC NPs exhibited excellent loading capacities, performed sustained and enzymatic release, and were stable in PBS, medium, and serum. After investigations *in vitro*, the DDC NPs were as effective as the dual drug combination in terms of cytotoxicity, antimigration, and apoptosis. Internalization results indicated that the DDC NPs could effectively deliver and fully release the payloads into PCA cells, and the process was mediated by the ligand-receptor interaction of HA with the CD44 protein. Low toxicity *in vivo* was confirmed by acute toxicity and hemolytic assays. The distribution *in vivo* showed that DDC NPs could enhance the accumulation of drugs in tumors and decrease nonspecific accumulation in normal organs. More importantly, DDC NPs significantly promoted the curative effect of the DOC and DOX combination in the PCA cell xenograft mouse model, indicating that the drugs with NPs did indeed act synergistically. This study suggests that the DDC NPs possess noteworthy potential as prospects for the development of PCA clinical chemotherapy.

## Introduction

In 2019, approximately 17,000 patients are expected to be diagnosed with PCA in the US, and such cases are approximately 20% of all new male cancers. PCA accounted for approximately 1 in 10 cancer-related deaths in 2018 (Siegel et al., 2019). Androgen deprivation has long been the first-line therapy for the disease; however, there was only an approximate 2–3-year survival time before the cancer progressed to an androgen-independent state (Bubendorf et al., 2000; Hellerstedt and Pienta, 2002; Roudier et al., 2003; Shah et al., 2006). Since the end of the last century, several studies have demonstrated that PCA cells can be effectively suppressed by mitotic spindle inhibitors such as paclitaxel and docetaxel (Beer and Raghavan, 2000; Canil and Tannock, 2004; Raghavan, 2004). Among these inhibitors, docetaxel is a derivative of taxane that enhances water solubility, reduces toxicity and broadens the antitumor spectrum. On this basis, docetaxel progressively became the first-line chemotherapeutic agent against PCA and increased the median overall survival of PCA patients (Liu and Zhang, 2013). However, the side effects of docetaxel cause excruciating suffering for many PCA patients and include myelosuppression, hepatotoxicity, and thrombosis, which caused most patients to discontinue using the drug (Singer and Srinivasan, 2012). Although several novel anti-PCA medicines, such as abiraterone and cabazitaxel, were approved by the FDA, the effect was still unsatisfactory (El-Amm and Aragon-Ching, 2013). Thus, the development of new treatments for PCA is of great importance and is in urgent practical demand.

Traditional antitumor chemotherapies, such as nontargeted and single drugs, are being slowly discontinued (Kahn et al., 2014). Combination chemotherapy has become a prospective successor in cancer treatment because the combination therapy has the synergistic effects of multiple drugs and has an impact on different action pathways to increase curative effects, decrease the necessary dosage, and reduce side effects (Broxterman and Georgopapadakou, 2005; Greco and Vicent, 2009). In the treatment of PCA, the combination of docetaxel and prednisone has been the standard clinical therapy since 2004 (Tannock et al., 2004). After decades of work, several combinations, including prednisone and satraplatin, epothilones and estramustine, have been developed for PCA clinical treatment (Pienta and Smith, 2005). Moreover, some combinations were investigated and evaluated in the laboratory phase; among them, the combination of paclitaxel and curcumin has attracted much attention. Several studies have reported that the combination of curcumin and paclitaxel could suppress PCA *via* various mechanisms, including downregulated expression of some proliferation factors and induced apoptosis (Wei et al., 2017). However, the current research on combination therapies could not satisfy the requirements for PCA treatment. Hence, the investigation and development of novel combination chemotherapies are still worthy endeavors. DOC remains the mainstream therapeutic agent for PCA treatment and is combined with other drugs, including mitoxantrone and estramustine, to treat PCA (Sinibaldi et al., 2002; Petrylak et al., 2004). Several clinical studies have demonstrated that DOC combined with anthracyclines could increase the anti-PCA effect because anthracyclines would enhance the sensitivity of the PCA cells to DOC (Pienta, 2001; Kouroussis et al., 2005; Mackler and Pienta, 2005; Neri et al., 2005; Petrioli et al., 2007; Neri et al., 2009). DOX is a kind of anthracycline that can prevent DNA remodeling (Pommier et al., 2010). Budman et al. (2002) have verified the synergistic effects of DOC and DOX in human PCA cell lines. Tsakalozou and colleagues further reported the synergistic effect of DOC combined with DOX in the treatment of human PCA cell lines (PC-3 and DU-145); they investigated various drug concentrations and proportions in their study (Tsakalozou et al., 2012). Nevertheless, there is an enormous obstacle to the further utilization of the DOX and DOC combination. The different physicochemical properties of these two drugs would cause differences in biodistribution and pharmacokinetic profiles. The difficulty in entering tumor tissues at the optimal dose and proportion fundamentally limits the synergistic effect of these drugs. The development of nanocarriers could effectively overcome the barriers to the delivery of multiple therapeutic agents (Hu and Zhang, 2012).

The nano vehicle encapsulates and delivers multiple drugs into tumors at the appropriate proportions and doses, which effectively decreases accumulation in normal organs and tissues to enhance the curative effects and minimize the side effects (Glasgow and Chougule, 2015). Numerous researchers have devoted themselves to the study of nanodelivery carrier use in PCA treatment and have obtained remarkable results. Sanna et al. (2011) prepared (-)-epigallocatechin 3-gallate nanocarriers with cross-linked targeting ligands on the surface to achieve targeted delivery through selective binding to prostate-specific membrane antigen (PSMA). The nanocarrier system exhibited an efficient targeting effect in PCA cell lines that express high levels of PSMA (Sanna et al., 2011). The team of Farokhzad has made a long-term commitment to the development of a nanocarrier system for chemotherapy. They used FDA-approved materials to design and prepare controlled-release NPs for DOC delivery that targeted PSMA (Farokhzad et al., 2006). Rocha and coworkers used polysaccharides to prepare nanoparticles for drug delivery targeted to PCA and demonstrated that the NPs could induce apoptosis in PCA cell lines (Rocha et al., 2011). Thangapazham and colleagues delivered curcumin *via* a targeted liposome with a surface that absorbed the PSMA antibody. These NPs effectively suppressed the proliferation of PCA cells (Thangapazham et al., 2008). In addition to chemotherapeutic agents, a gene was also delivered by nanoparticles. Peng et al. (2007) used polymeric NPs to deliver the diphtheria toxin suicide gene into PCA cells and thus significantly inhibited the progression of PCA. In recent years, extracellular vesicles (EVs), such as exosomes, have been revealed to be ideal candidates for drug delivery because the EVs can interact with related target cells in local or distant areas (Fais et al., 2016). EVs have been used to encapsulate small molecular agents, oncolytic viruses, in the treatment of various tumors (Yang et al., 2013; Pascucci et al., 2014; Ran et al., 2016; Garofalo et al., 2018; Garofalo et al., 2018). In the treatment of PCA, Saari et al. (2015) used EVs that effectively enhanced the cytotoxicity of Paclitaxel in PCA cells. In a previous study, our group developed nanocarriers for the encapsulation of dual drugs useful for antitumor treatment. The NPs were coloaded with DOX and apogossypolone and were adjustable in terms of drug dose and ratio. Moreover, the outer material was comprised of HA, which could provide a tumor target. In that study, tumor suppression was evaluated *in vivo* in a PC-3-bearing mouse model. The NPs effectively enhanced the inhibition of tumor progression in the mice, with relatively few side effects (Li et al., 2015).

In the present study, multifunctional nanocarriers were used to overcome the pharmacokinetic differences between DOX and DOC and to achieve maximal anti-PCA effects and minimal side effects. Initially, the synergistic anti-PCA effect of DOX and DOC was evaluated by cytotoxicity assay in three human PCA cell lines (PC-3, DU-145, and LNCap), which verified the optimal ratio of the two drugs. CSaSt and HA were then used for the coencapsulation of DOC and DOX through self-assembly methods, thereby creating the DDC NPs. When the DDC NPs had been prepared and characterized, three human PCA cell lines were used for the evaluation of internalization and inhibition *in vitro*, and mouse models were used to investigate delivery and suppression *in vivo*. The study improved the synergistic effect of DOC and DOX in an anti-PCA treatment and demonstrated that there is an excellent outlook for the use of DDC NPs in PCA therapy.

## Materials and Methods

### Materials

Human PCA cell lines (PC-3, DU-145, and LNCap) were provided by Sure Bio-Tech Co., Ltd. (Shanghai, China). The cells were cultured in DMEM containing 10% fetal bovine serum (FBS) and 1% antibiotics. The medium, trypsin, and antibiotics were purchased from HyClone Co., Ltd. (UT, USA). The FBS was obtained from Gibco Co. (NY, USA). DOX, DOC, HA, coumarin-6, and IR-780 were purchased from Aladdin Corp. (Shanghai, China). The protein extraction kit, CCK-8 kit, TUNEL staining kit, and cell apoptosis detection kit were purchased from Beyotime Co., Ltd. (Shanghai, China). The antibodies (Bcl-2, Bax, Caspase 3, horseradish peroxidase-labeled secondary antibody) were supplied by Cell Signaling Co. (MA, USA). Other dyes and chemical reagents were obtained from Bokeri Co., Ltd. (Xi’an, China). The BALB/c mice and BALB/c-nu/nu mice were provided by Peking HFK Biotech Co., Ltd. (Beijing, China). CSaSt was synthesized in our lab.

### Optimal Synergistic Proportions of the Drugs

To obtain the optimal proportions of DOX and DOC, CCK-8 assay was used for the detection of suppression in different combinations *in vitro*. Three PCA cell lines (PC-3, DU-145, and LNCap) were cultured in complete DMEM high glucose medium (10% FBS and 100 U/mL of antibiotics) at 37°C under 5% CO_2_ in an incubator (MCO-20AIC, SANYO, Osaka, Japan). The cells were seeded in 96-well plates at a concentration of 0.8×10^4^ cells/well. After 24 h, the treatment was applied. The DOX concentration was 100-800 nmol/L, and the DOC concentration was 0.25-2 nmol/L. All of the treatments were repeated in three wells during the experiment. After 48 h, 100 μL of colorless DMEM, which contained 10% CCK-8 (v/v), was used to replace the stale medium in each well, where it continued to incubate for 2 h. The optical density (OD) of wells at 450 nm was then measured with a microplate reader (Infinite^®^ 200 Pro, Tecan, Switzerland). The IC_50_ values were calculated by GraphPad Prism 5.0 software. According to the IC_50_ values, the cells were treated with different combinations of DOX and DOC. The combination index (CI) was then computed with CompuSyn software (Liu et al., 2005). The optimal drug proportions for effective synergy were ascertained based on the CI values, where a lower CI value meant a better synergistic effect.

### Construction of DDC NPs

For the preparation of DDC NPs, we refer to the research of Li et al. (2015). Initially, the DOC micelles were constructed with CSaSt and DOC *via* hydrophobic interactions. DOC and CSaSt were codissolved in DMSO at ratios of 1:8 to 1:10. Subsequently, the DMSO solution was added dropwise into water while stirring. After 10 min of stirring, the DOC MC solution was obtained. In addition, the DOX NPs were directly prepared by DOX and HA *via* electrostatic interactions. The proportion of HA to DOX was more than 10:1, and overdoses of HA were ensured. The DOC MC solution was then injected dropwise into the DOX NPs solution at the appropriate drug ratio. After 20 min of stirring, the fabrication of the DDC NPs was completed. DMSO, unencapsulated DOX, and other soluble impurities were removed by dialysis. The surplus materials were removed by centrifugation.

### Detection of DDC NPs Properties

The DDC NPs morphology was examined by transmission electron microscopy (TEM) (JEM-2100F, JEOL, Japan). The size and zeta potential were measured with a Malvern instrument (Nano-ZS90, Malvern, UK). The concentrations of DOX and DOC were detected by fluorescence spectrophotometry and HPLC, respectively. The loading capacity was then calculated on the basis of the concentration data.

The release of DOX in the DDC NPs *in vitro* was measured by dialysis. Free DOX was used as a control. HAase was added for the enzymatic released test. All samples contained the same concentration of DOX and were dialyzed under the same conditions. The direct release of DOC was too difficult to measure because the drug was practically insoluble in water. Thus, the indirect release ratio of DOC was investigated by maintaining the amount of DOC during lyophilization after dialysis.

The stability of the DDC NPs was measured on the basis of changes in size under different conditions. To ensure that the DDC NPs could be used in subsequent experiments *in vitro* and *in vivo*, PBS, complete medium, and FBS were used as dispersed solutions.

### Cytotoxicity of DDC NPs *In Vitro*

In order to demonstrate that DDC NPs has equivalent inhibitory effect with free drugs, three human PCA cell lines (PC-3, DU-145, and LNCap) were used for the evaluation of the cytotoxicity test *in vitro*. The cells were seeded into 96-wells plates (densities of cell lines: PC-3: 0.6×10^4^ cells/well, DU-145: 0.8×10^4^ cells/well, LNCap: 0.8×10^4^ cells/well) and incubated at 37 °C with 5% CO_2_ for 24 h. The cells were then treated with complete DMEM medium with DDC NPs, and the free drugs in combination at the same concentrations as used in the DDC NPs were used as controls. The concentration gradients in DOC were 0.25, 0.5, and 1 nmol/L, and those in DOX were 100, 200, and 400 nmol/L. After 48 h of treatment, the cytotoxicity was also evaluated by a CCK-8 assay, as described previously.

### Clone Formation Assay

The level of inhibited cell proliferation *in vitro* was evaluated by a clone formation assay. The PC-3 cells were seeded into 60 mm dishes at a density of 500 cells/dish and incubated with complete medium containing DDC NPs, dual drugs, DOX, and DOC. The concentration of DOX was 200 nmol/L, that of DOC was 0.5 nmol/L, and dual drugs consisted of 200 nmol/L of DOX added to 0.5 nmol/L of DOC. The concentrations of the drugs in the DDC NPs group were equal to those in the dual drug group. All dishes were incubated at 37 °C with 5% CO_2_ for 72 h. The stale medium was then replaced by fresh medium with 20% FBS. After 5 d, the cells were fixed with 4% paraformaldehyde and stained with crystal violet. The number of clones in each group was quantified.

### Transwell Assay

Transwell assays were used to evaluate cell migration after different treatments. Millicell hanging cell culture inserts (8.0 μm, Millipore, Darmstadt, Germany) were used to perform the transwell assay. Samples of 200 μL of FBS-free medium with 4×10^4^ PC-3 cells, which were treated with DDC NPs, dual drugs, DOX, and DOC, respectively, were then added into the upper chamber of the insert. The medium with 40% FBS was added to the 24-well plate. The insert was then placed in the well of the plate, and the bottom of the insert was immersed into the medium. The plate was incubated at 37 °C with 5% CO_2_ for 24 h. Subsequently, the inserts were steeped with methanol and stained with crystal violet. The cells remaining in the upper chamber were removed. The inserts were then examined by microscopy (DP72, Olympus, Tokyo, Japan), and the number of migrated cells was counted in five random fields.

### Apoptosis Investigation

The extent of apoptosis was investigated by flow cytometry and western blotting. In the flow cytometry assay, the PC-3 cells were seeded into 10 mm dishes and cultured to 60-70% confluence. The cells were then treated with DDC NPs, DOX, DOC, or dual drug combinations. The concentration of DOX was 100 nmol/L, that of DOC was 0.25 nmol/L, and the dual drugs contained 100 nmol/L of DOX added to 0.25 nmol/L of DOC. The concentrations of the drugs in the DDC NPs group were equal to those in the dual drugs group. After 48 h of treatment, the cells were stained with Annexin V/PI and detected by flow cytometry (C6, BD Accuri^®^, NJ, USA).

The PC-3 cells were seeded into 10 mm dishes and cultured to 60-70% confluence. A DDC NPs sample, DOX, DOC, or dual drug combination was added to the dishes. After 24 h, the proteins were extracted with a protein extraction kit and quantified by BCA assay. The samples were separated by 12.5% SDS-PAGE gel electrophoresis and transferred to polyvinylidene fluoride membranes. The membranes were blocked with 5% BSA solution and incubated with primary antibodies (against Bcl-2, Bax, Caspase 3, and β-actin). Subsequently, the membranes were incubated with a second antibody, and the bands were visualized by using an ECL Plus system (Thermo Fisher Scientific, Waltham, MA, USA). The intensity of the band was analyzed by ImageJ software. The intensity of the β-actin protein served as a loading control.

### Cell Internalization and Affinity of DDC NPs *In Vitro*

The green fluorescent dye coumarin-6 was used to label the NPs. The encapsulation process of coumarin-6 was the same with DOC; thereinto, the dye and CSaSt were encapsulated in the NPs at ratios of 1:8 to 1:10. Moreover, DOX has red fluorescence. The NPs were therefore visualized with dual fluorescence, which was examined to determine internalization and affinity. The PC-3 cells were seeded into φ3.5 mm confocal cell dishes. When the cells had been cultured to 40-50% confluence, the fluorescent NPs were co-incubated with PC-3. The cells were fixed with 4% paraformaldehyde solution at sequential time points, and the nuclei were stained with a DAPI kit. A confocal microscope (TCS SP5 II, Leica, Germany) was used to examine the dishes. The cell affinity test was aimed at investigating whether endocytosis in the cells with NPs was mediated by HA. HA was utilized to block endocytosis by competition. The PC-3 cells were cultured in complete medium containing 1% HA for 24 h, and then fluorescent NPs were added. The cells in normal medium were used as the control. After incubation, the cells were treated and observed. The fluorescence intensities were determined and quantified by ImageJ software.

### Acute Toxicity *In Vivo*

Ten male and ten female BALB/c mice, which each weighed approximately 18 g, were randomly distributed into two groups. The mice were adapted to the breeding environment for 5 d and labeled with ear tags. The mice were then intravenously injected with the dual drug combinations or DDC NPs at the same dose (DOX: 20 mg/kg and DOC 0.05 mg/kg). The death rate of mice in each treated group was calculated after 2 w. The live mice were euthanized by CO_2_ overdose, and the organs were collected for pathological evaluation. In this study, all animal experiments were conducted following the Guidelines for the Use and Care of Experimental Animals at Xi’an Medical University and were approved by the Laboratory Animal Administration Committee of Xi’an Medical University. The Animal Ethics Approved Document Number is XY-AUC-2017-213.

### Hemolysis Assay

The whole blood of the mice was collected and immediately treated with heparin. The blood was divided into six pools that were treated with Triton X-100 (1% v/v), DMSO (0.5% v/v), a dual drug combination (DOX: 20 mg/ml, DOC: 0.05 mg/ml), DDC NPs (concentrations were the same as those in the drug combination), empty NPs, or saline. The samples were incubated in a 37°C water bath for 2 h and were subsequently centrifuged at 13,000 rpm for 15 min. The level of hemolysis was evaluated on the basis of the absorbance of the supernatant at 394 nm.

### Xenograft Mouse Model

Male BALB/c-nu/nu mice (4 weeks old with an average weight of approximately 16 g) were fed in the SPF breeding room for adaptation. After 7 d, the physiological status of the mice was evaluated. Then, 100 μL of PC-3 cell suspension, at a density of 5×10^6^ cells/mL, was injected into the inguinal area of each mouse. After 5 d, the mice were checked daily. When the tumor volumes had increased to an appropriate range, the xenograft models were used for experiments *in vivo*.

### Delivery Evaluation *In Vivo*

The NIF fluorescent dye IR780 was used to label the DDC NPs. Both DOC and IR-780 are hydrophobic compounds; therefore, IR-780 was encapsulated in core micelles to replace DOC. Two xenograft animal models were selected for investigation of the delivery of DDC NPs *in vivo*. Two hundred microliters of NIR-labeled NPs with 20 μg/mL of IR-780 and 200 μL of an equal concentration of IR-780 solution were intravenously injected into two mice. Fluorescence imaging was used to measure the signal distribution *in vivo* with an IVIS imaging system (Perkin Elmer, Waltham, MA, USA). At the end of the experiment, the mice were euthanized by CO_2_ overdose, and the organs and tumor tissues were collected for fluorescence observation.

### Antitumor Investigation *In Vivo*

Thirty PCA xenograft mouse models were prepared for the antitumor experiment *in vivo*. When the average tumor volume had reached approximately 200 mm^3^, the mice were randomly divided into five groups. The treatments in the groups were saline, DOX separately, DOC separately, DOC and DOX combination, and DDC NPs. The samples were administered at the same dose *via* intravenous injection. The injection volume was 200 μL, and the injection frequency was twice per week. The tumor sizes and body weights were recorded during the experiment. After 3 weeks, the mice were euthanized by CO_2_ overdose. The tumor tissues were collected and weighed. The tissues were then fixed with 4% paraformaldehyde. Histological investigation was performed by paraffin section *via* HA staining and TUNEL staining.

### Statistical Analysis

The Student t-test and one-way and two-way ANOVA were utilized in the analysis of the data *via* GraphPad Prism 5.0 software. The data are presented as the mean ± SD of independent experiments. A P-value < 0.05 indicated that the data showed a significant difference.

## Results

### The Optimal Synergistic Proportion of DOX and DOC

**Figure 1:**
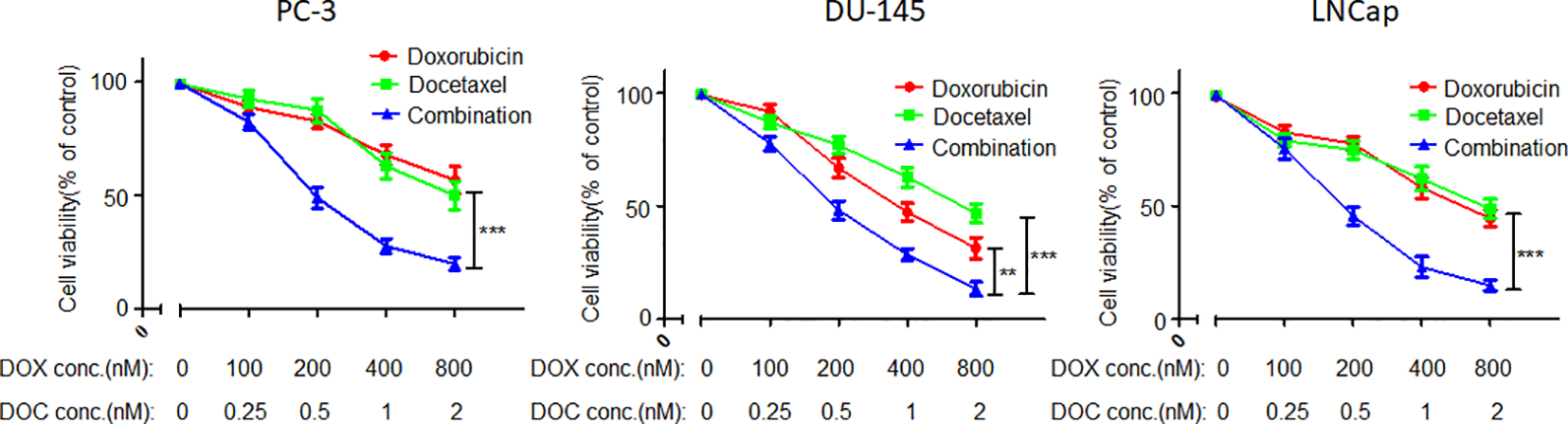
Inhibitory effects of DOC, DOX, and their combination in three PCA cell lines. Error bars represent the SD of the mean. P-values in the results were calculated by Tukey’s post-test; ** indicates P < 0.01, *** indicates P < 0.001.

As shown in a previous report, DOX and DOC have synergistic effects against PCA (Tsakalozou et al., 2012). In the present study, the synergy of DOX and DOC was further demonstrated by CCK-8 assay. More importantly, the optimal proportion of DOX and DOC was obtained *via* the experiment. As **Figure 1** shows, the combination with a DOX to DOC ratio of 400:1 exhibited the most effective suppression of proliferation in all three PCA cell lines. The cell viability in the combination treatment group was significantly lower than that of the two single drug-treated groups. Moreover, the two drugs coencapsulated in the NPs could be delivered at this proportion.

### Preparation and Characterization of DDC NPs

**Figure 2:**
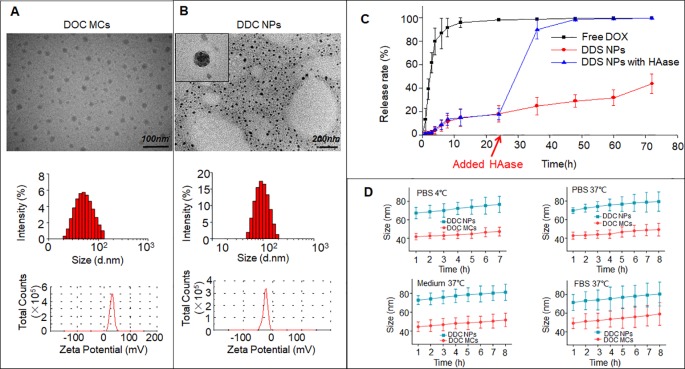
Characteristics of DDC NPs. TEM photos, size, and zeta potential of DOC MCs **(A)** and DDC NPs **(B)**. *In vitro* release of DDC NPs **(C)**. *In vitro* stabilities of DDC NPs in PBS, medium, and FBS **(D)**.

Initially, DOC was encapsulated by CSaSt and formed MCs with a size of 45 ± 4.5 nm and zeta potential of 31 ± 2.1 mV (**Figure 2A**). The DOX NPs, which were 12 ± 3.7 nm in size and had a zeta potential of -25 ± 1.9 mV, were absorbed around the surface of the DOC MCs. Finally, the DDC NPs were prepared. As shown in **Figure 2B**, the TEM revealed that the morphology of the DDC NPs that of spherical particles with rough surfaces. The size was 68.4 ± 7.1 nm, and the zeta potential was -22 ± 2.2 mV. The size and zeta potential results further demonstrated that during the process of construction, DOX NPs were absorbed on the surface of DOC MCs *via* electrostatic interactions.

### Encapsulation, Release and Stability of DDC NPs

The encapsulation capacities assessed included EE and DL. The DOX data were measured using a fluorescence spectrophotometer, and the results were 91.4 ± 3.7% and 9.1 ± 1.4%, respectively. DOC was measured by HPLC; the EE was 96.1 ± 2.3%, and the DL was approximately 0.02%. The proportions of the DOX and DOC aligned with previous results, which meant that the DDC NPs could achieve effective synergy.

The release of the drugs *In vitro* was evaluated by DOX dialysis. The release curves are shown in **Figure 2C**. In the free DOX group, the burst releasing phenomenon was extremely obvious. Approximately 90% of the DOX was released to the outside of the dialysis membrane in the initial 8 h. After 12 h, less than 5% of the DOX remained in the dialysis membrane, and DOX almost reached the outer phase in another 12 h. In contrast, the DOX in the DDC NPs group showed gradual release within 72 h, and nearly 60% of the DOX remained in the dialysis membrane at the end of dialysis. HAase was used for the investigation of enzymatic release. As shown in the results, before the addition of HAase, the release curve was smooth. However, approximately 70% of DOX was released within 12 h when HAase was added. In contrast, DOC is insoluble in water; hence, the release was indirectly evaluated by the residual amount after dialysis. The results indicated that the vast majority of the DOC remained in the DDC NPs after dialysis. The stability was reflected by changes in the hydrodynamic diameter. As **Figure 2D** shows, at 4°C, both the DDC NPs and the DOC MCs exhibited good stability in PBS, and when the temperature was increased to 37°C, the sizes of the DDC NPs and DOC MCs were also unchanged. To verify that the DDC NPs could be used in subsequent experiments, the NPs were dispersed into complete medium and FBS. At 37°C, the sizes of the DDC NPs and DOC MCs exhibited a slight increase. However, over time, the DDC NPs and DOC MCs ceased to swell. The size increased by approximately 10%.

### Cytotoxicity of the DDC NPs *In Vitro*

**Figure 3:**
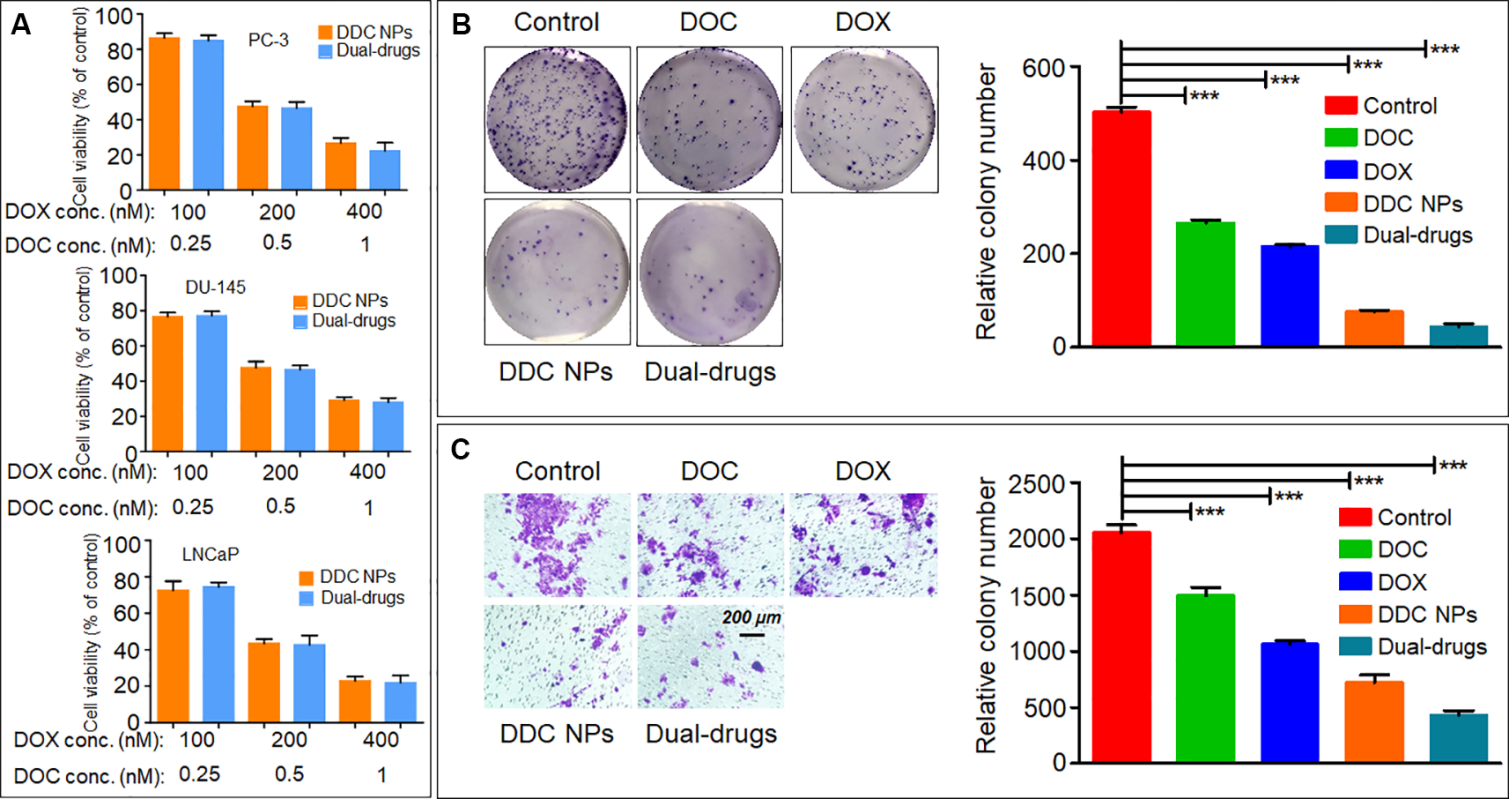
*In vitro* inhibition of DDC NPs. Cytotoxicity of DDC NPs in three PCA cell lines. The cells were treated by DDC NPs and dual drugs with different concentrations for 48 h. **(A)**. The results of clone formation assay of DDC NPs **(B)**. The results of transwell assay of DDC NPs **(C)**. Error bars represent the SD of the mean. P-values in **(B** and **C)** were calculated by Bonferroni’s post-test of ANOVA; *** indicates P < 0.001.

The DDC NPs efficiently retained the synergistic effect of DOX and DOC. As shown in **Figure 3A**, the DDC NPs exhibited the same inhibitory effect in all three PCA cell lines as the combination of free drugs of equal concentrations.

The clone formation assay further demonstrated that DDC NPs can effectively suppress proliferation. The PC-3 cells were used in this test. The results are shown in **Figure 3B**. The clone number in the DDC NPs-treated group was much less than it was in the single drug treatment and saline groups, and it was not significantly different from the dual-drug combination.

### Antimigratory Effects of DDC NPs *In Vitro*

Transwell assay was employed to evaluate the antimigratory effect of the DDC NPs. The PC-3 cells were also used for this experiment. The results are shown in **Figure 3C**. It is evident that the DDC NPs can inhibit cell migration. The effect was similar to that of the clone formation assay. The transmembrane cells in the DDC NPs-treated group were significantly decreased compared with those in the single drug-treated groups. In addition, the inhibition of the dual drug combination treatment was also similar to that of the DDC NPs.

### Evaluation of Apoptosis

**Figure 4:**
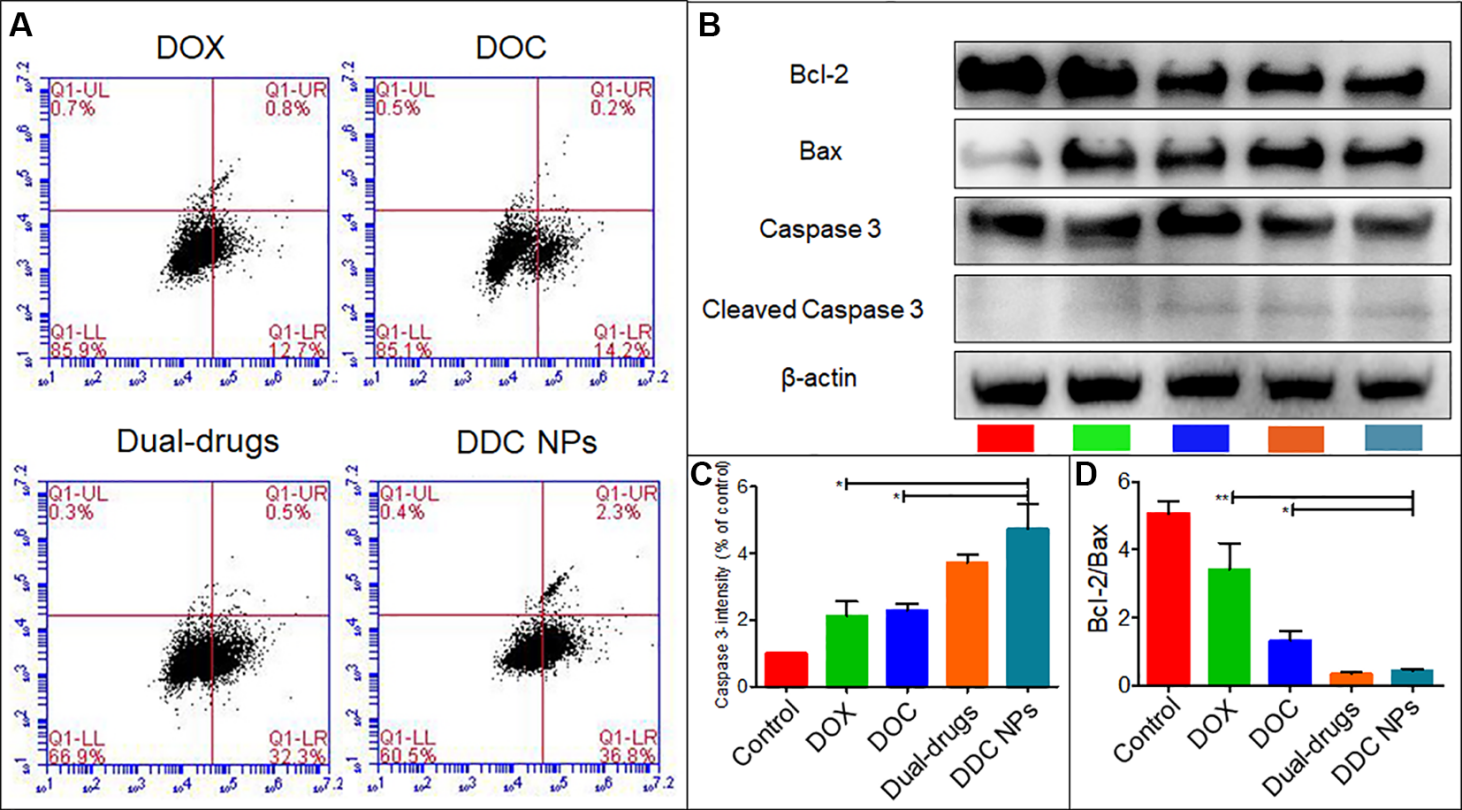
Induction of apoptosis. Results of flow cytometry **(A)**. Western blotting of apoptosis-related factors **(B)**. Quantitative analysis of Bcl-2/Bax and cleaved Caspase 3 **(C** and **D)**. Error bars represent the SD of the mean. P-values in B and C were calculated by Bonferroni’s post-test of ANOVA; * indicates P < 0.05, ** indicates P < 0.01.

Whether the DDC NPs mechanism involves triggering apoptosis was first determined through flow cytometry analysis. As shown in **Figure 4A**, early apoptosis was clearly observed in the DDC NPs-treated group and the dual drug combination-treated group. By contrast, both of the single drug-treated groups exhibited a significantly lower apoptosis ratio. Moreover, the results from the western blot analysis further verified that the DDC NPs could induce apoptosis. As shown in **Figures 4B**–**D**, Bcl-2, Bax, and cleaved Caspase 3 were affected by the treatments. The expression of Bcl-2, which is an antiapoptotic protein, was decreased in DDC NPs and dual drug combinations. The ratio of Bcl-2 and Bax in the DDC NPs was significantly lower than it was in the single drug-treated groups and the saline-treated group. Moreover, the pro-apoptotic factor cleaved Caspase was obviously increased in the DDC NPs-treated group. The results verified that DDC NPs could effectively induce apoptosis.

### Internalization and Affinity of the DDC NPs *In Vitro*

**Figure 5:**
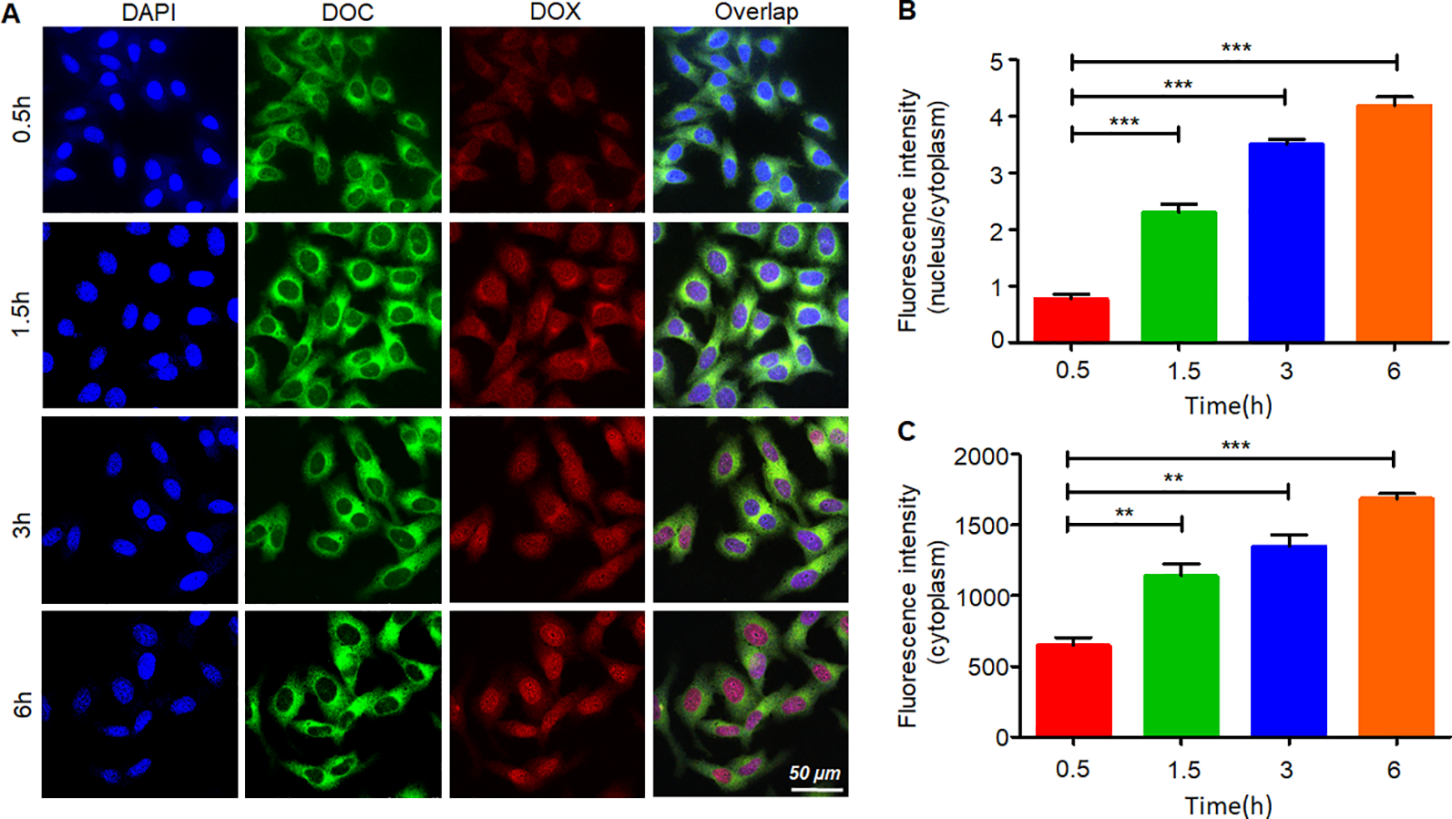
Internalization and intracellular release of DDC NPs. Confocal imaging of the fluorescent-labeled DDC NPs **(A)**. Quantitative analysis of fluorescent intensity in cells **(B** and **C)**. Error bars represent the SD of the mean. The means were compared using one-way ANOVA; ** indicates P < 0.01, *** indicates P < 0.001.

**Figure 5** shows the process of internalization. The green fluorescence was emitted by coumarin-6. The dye mainly stains the cell membrane and primarily accumulates in the cytoplasm. DOX emits red fluorescence, and it binds to the nucleus. The change in the distribution and intensity of the fluorescence signal could reflect the process of internalization and intracellular release. In the first 30 min, both the green and red fluorescence signals were concentrated in the cytoplasm, and the fluorescence intensities were relatively weak. Then, over time, the fluorescence intensity increased, and the signals separated. DOX started to gather into the nucleus. After 3 h, the intensity of the red fluorescence signal had accumulated equally in the cytoplasm and the nucleus. At the 6^th^ hour, almost all the DOX had accumulated in the nucleus, and coumarin-6 remained in the cytoplasm. These results indicated that the DDC NPs could effectively deliver the payloads into the cell, where they were fully released.

**Figure 6:**
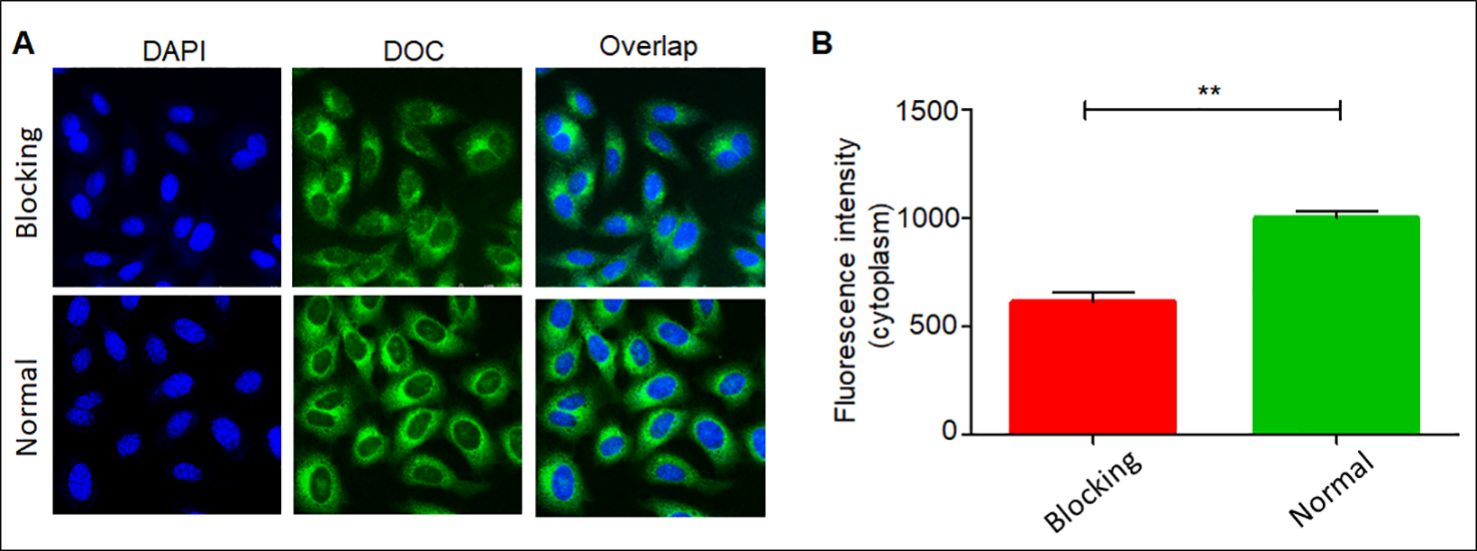
Affinity of DDC NPs in PCA cells. Confocal imaging of DDC NPs with/without HA blocking **(A)**. Quantitative analysis of fluorescent intensity in cells **(B)**. Error bars represent the SD of the mean. ** indicates p < 0.01 in t-test.

The cell-targeted delivery of the DDC NPs depended on ligand-receptor mediation *via* HA and CD44. The HA blocking test was used to investigate the mediated endocytosis. As shown in **Figure 6**, the fluorescence intensity in the HA pretreated group was significantly lower than that in the control group, suggesting that HA competitively suppressed the endocytosis of the DDC NPs in the PC-3 cells. Thus, the results indicated that the DDC NPs could be used for tumor-targeted delivery *in vivo*.

### Acute Toxicity *In Vivo*

**Figure 7:**
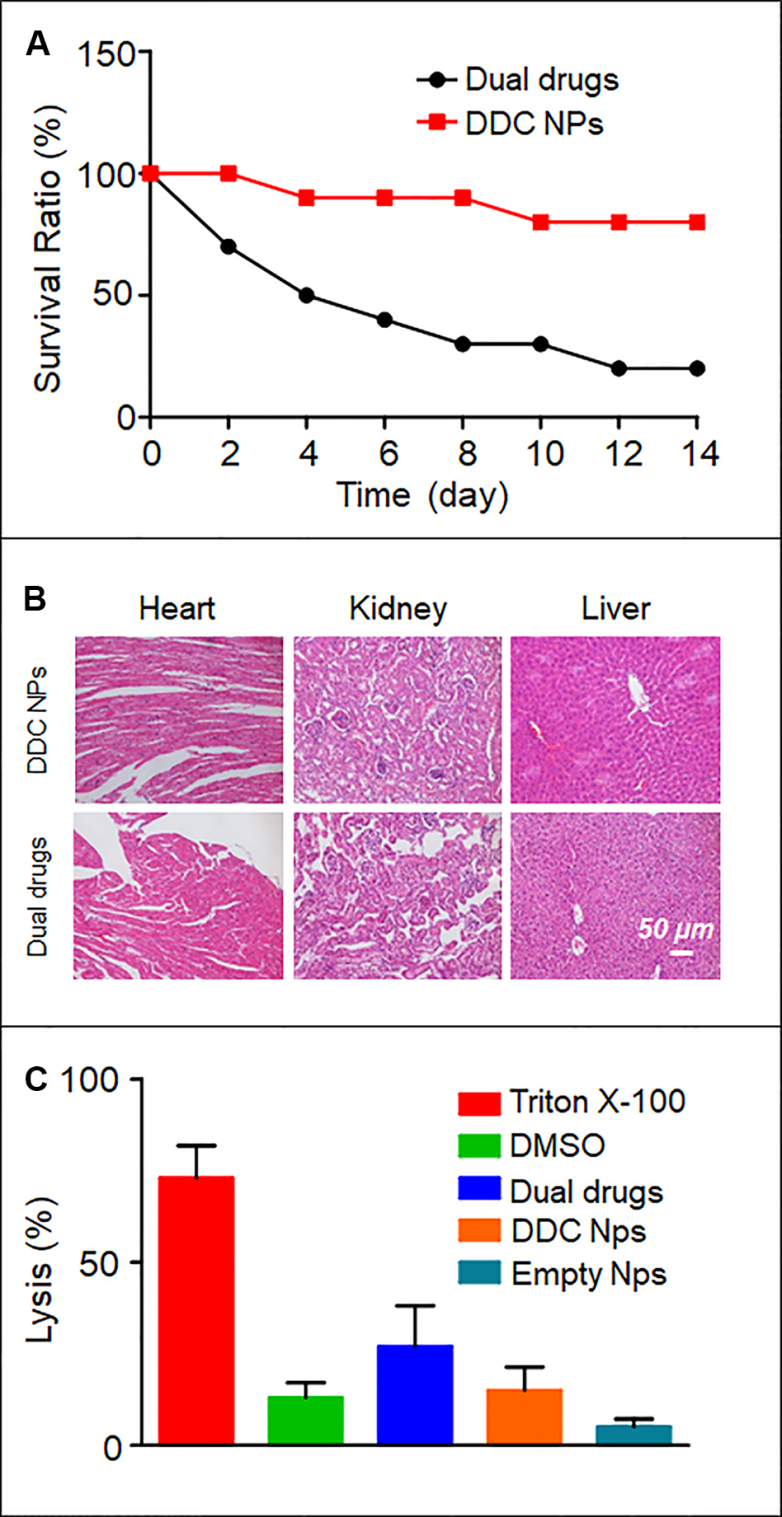
*In vivo* toxicity of DDC NPs. Mouse survival rate in acute toxicity **(A)**. Pathological sections of heart, liver, and kidney **(B)**. Results of hemolytic test **(C)**.

Toxicity *in vivo* is the primary obstacle to using chemotherapeutic agents. In the present study, an acute toxicity test was used to evaluate whether the DDC NPs could decrease the toxicity induced by the drugs *in vivo*. The results are shown in **Figure 7A**. At the same dose of drugs, the mortality in the DDC NPs-treated group was significantly lower than that in the dual drug combination group. After 14 d, only 20% of the mice had survived in the dual drug combination group; by contrast, 80% of the mice in the DDC NPs-treated group had survived. The pathological results further demonstrated that the DDC NPs could decrease the toxicity in other organs. The results are shown in **Figure 7B**. In the dual drug combination groups, the cardiac tissues exhibited characteristics typical of myocarditis, which included disappearing myocardial cells, extravasated blood and inflammatory cell infiltration. In addition, pathological changes occurred in the kidney. Several glomeruli were observed through acidophilic staining, and the small vessels in mesenchyme exhibited dilation and congestion. Moreover, injuries were detected in hepatic tissue. Hepatocytes exhibited irregular arrangement, swelling, and accumulated lipid droplet vacuoles. In contrast, the tissues from the DDC NPs-treated group did not show serious pathological changes.

### Hemolytic Test

The results of the hemolysis assay are shown in **Figure 7C**. As the positive control, Triton X-100 caused severe hemolysis, with a lysis rate of approximately 70%. More than 10% of the DMSO-treated sample had lysed. Treatment with dual drug combinations caused nearly 30% the cells to undergo hemolysis. The hemolysis ratios in the DDC NPs-treated group and the empty NPs group were 15% and 5%, respectively. These results suggested that the DDC NPs could effectively decrease the damage to erythrocytes caused by drugs.

### Targeted Delivery *In Vivo*

**Figure 8:**
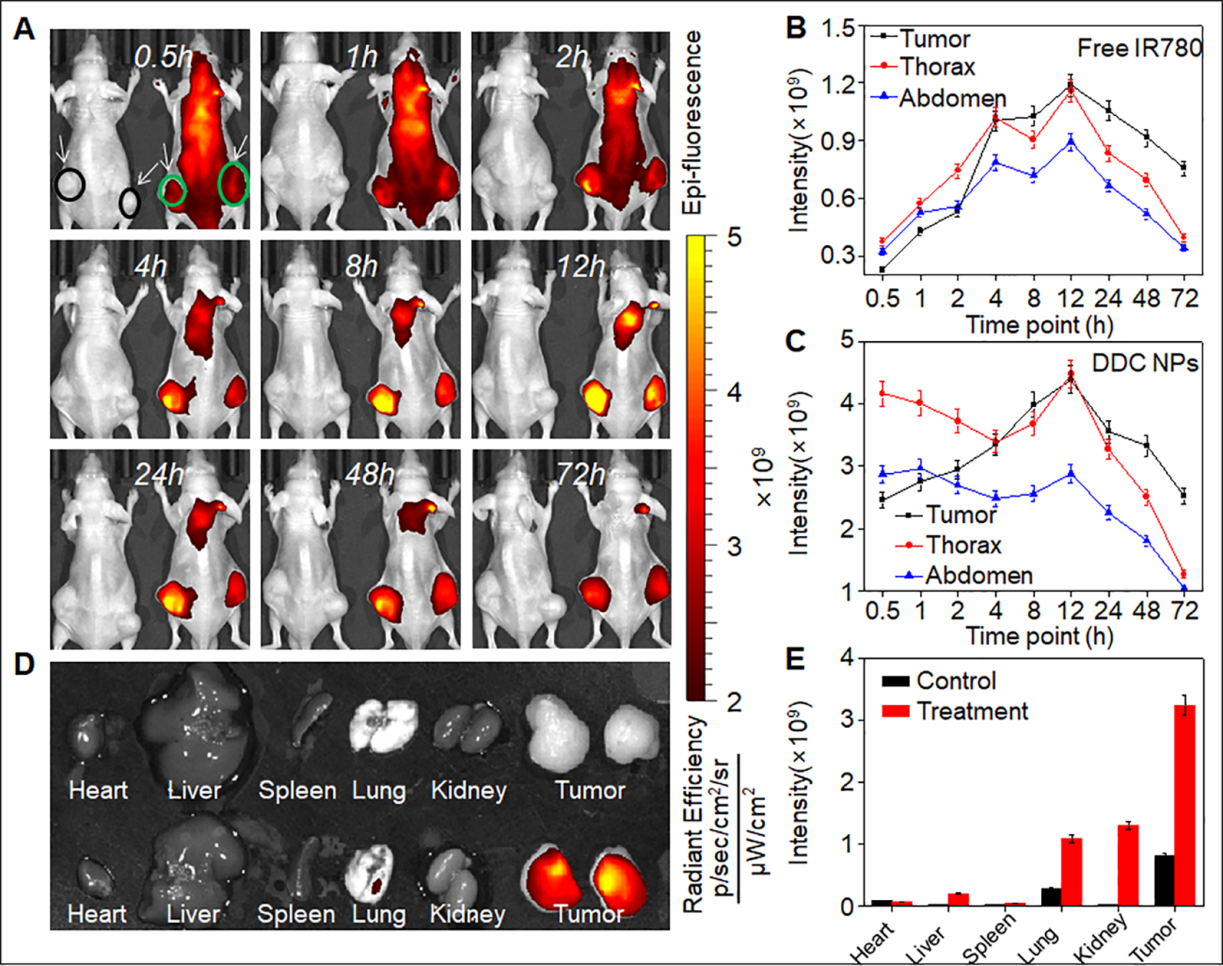
*In vivo* targeted delivery of DDC NPs. Fluorescent signal distribution of DDC NPs during the experiment **(A)**. Quantitative analysis of fluorescent intensity in mice **(B** and **C)**. Fluorescent intensity in organs and tumors **(D)**. Quantitative analysis of fluorescent intensity in tissues **(E)**.

Fluorescence imaging was employed *in vivo* to investigate the distribution of the DDC NPs in a PCA xenograft mouse model. The NIF fluorescent dye IR-780 was used for labeling the NPs. Two mice were used in this experiment. One mouse (left) was injected with free IR-780, and the other mouse (right) was treated with DDC NPs (**Figure 8A**). The tumors are marked by arrows. After 30 min of injection, the difference in the fluorescence signal between the two mice was obvious. As shown in **Figures 8B** and **C**, the signal in the mouse injected with free IR-780 was significantly lower than that in the DDC NPs-treated mouse, and the total intensity was approximately 10-fold different. In the free IR-780-injected mouse, the distribution of fluorescence did not exhibit any obvious difference among the main parts of the body. However, in the DDC NPs-injected mouse, the fluorescence mainly accumulated in the thorax and abdomen within the first 1 h. After 2 h, the fluorescence signal in the tumor areas gradually increased and peaked at 12 h. Although the fluorescence was also largely concentrated in the thorax, the signal attenuation was faster in the thorax than in the tumor area. After 72 h, the fluorescence in the thorax and abdomen was significantly lower than it was in the tumor area. The fluorescence distribution in tissues (**Figure 8D**) further demonstrated that the DDC NPs could effectively deliver to and accumulate in tumors. As shown in **Figure 8E**, in the DDC NP-injected mouse, the intensity of the signals in the tumors was obviously higher than it was in the organs. The results indicated that the DDC NPs possessed excellent tumor-targeted effects. But meanwhile, the results also indicated that the tumor accumulation was high 8-48 h after injection. Because the main materials of the DDC NPs were starch and HA, they had a short half-life *in vivo*. Thus, the injection interval was not more than three days in the tumor inhibition experiment.

### Tumor Suppression *In Vivo*

**Figure 9:**
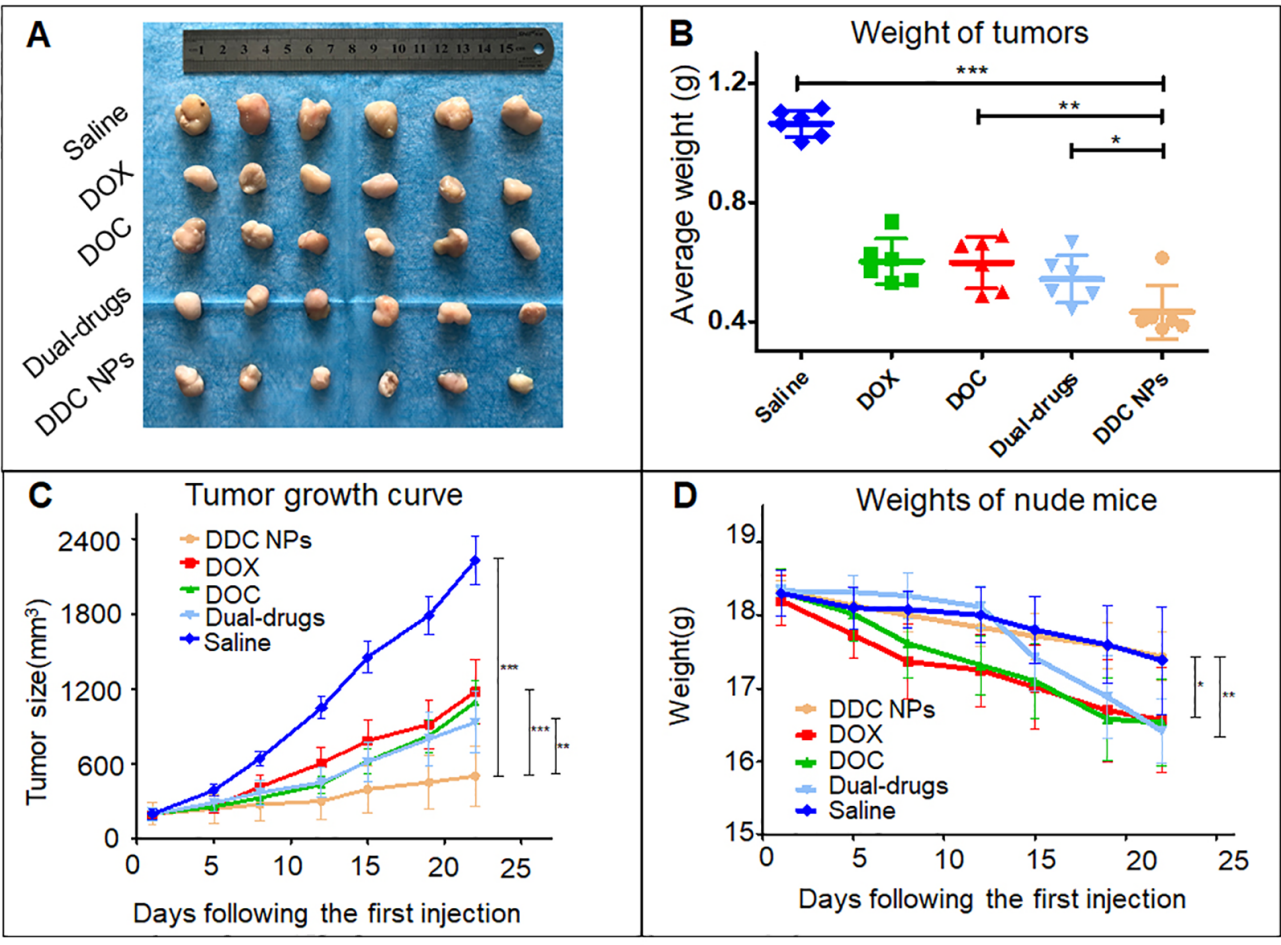
*In vivo* antitumor effect of DDC NPs. Photograph of tumor tissues **(A)**. Weight of tumors **(B)**. Tumor growth curves in treated groups **(C)**. Changes in body weight in each group **(D)**. Error bars represent the SD of the mean. P-values in the results were calculated by Tukey’s post-test; * indicates p < 0.05, ** indicates P < 0.01, *** indicates P < 0.001.

The PC-3 cell xenograft mouse models were used to evaluate tumor suppression of DDC NPs *in vivo*. The doses of DOX and DOC were approximately 2 mg/kg and 5 μg/kg, respectively, which aligned with the optimal proportion obtained in the previous experiment. The DDC NPs treated group and the combination treated group have same doses of the drugs. As shown in the tumor photos and tumor growth curves (**Figures 9A**, **C**), the average volume of the tumors in the saline-treated group increased more than 10-fold during the experiment. The free drug treatments inhibited tumor progression to a certain extent; however, the inhibitory effects were unsatisfactory. The average tumor volume in the DOX-, DOC- and dual drug-treated groups increased approximately 6-, 6-, and 5-fold, respectively. It is noteworthy that the DDC NPs exhibited excellent antitumor effects. The average tumor volume increased only approximately 2-fold, and the tumors were significantly smaller than those in other treatment groups. The result was further demonstrated by differences in tumor weights (**Figure 9B**). The tumors in the DDC NP-treated group were obviously lighter than the tumors in the free drug-treated and saline-treated group. **Figure 9D** presents changes in body weight, illustrating the differences between groups, and shows the weight change during the experiment. The mice in the free drug-treated groups exhibited an obvious decrease. However, there were no significant differences between the DDC NP-treated group and the saline-treated group. The results indicated that the DDC NPs could effectively perform such that the synergistic effects of DOX and DOC were realized with few side effects *in vivo*.

### Histological Examination

**Figure 10:**
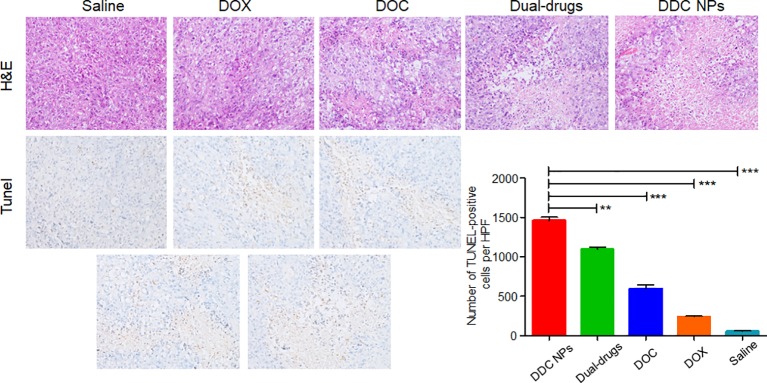
Histological assay of tumors. Photographs of HE staining and TUNEL staining. Quantitative analysis of positive spots in TUNEL assay. P-values in results were calculated by Bonferroni’s post-test of ANOVA; ** indicates P < 0.01, *** indicates P < 0.001.

The pathological sections were used to investigate the antitumor effect at the histological level. The top row in **Figure 10** shows HE staining of tumor tissues in all five groups. The treated groups exhibited necrosis to a certain extent. Among them, tumors in the DDC NPs group showed the most severe necrosis. The necrosis in the tumors of the other treated groups was indicated with lighter dye, and a few pathological lesions were found in the saline groups. The results of the TUNEL staining are shown in the bottom images in **Figure 10** and shows significant differences. The tumors in the saline group had rare positive spots (brown). The free drug-treated groups exhibited partially positive spots. In contrast, positive spots appear extensively as dots in the DDC NP-treated groups. TUNEL staining can effectively reflect cell death in tumor tissues. These results indicated that treatment with DDC NPs could synergistically inhibit the progression of PCA.

## Discussion

**Scheme 1:**
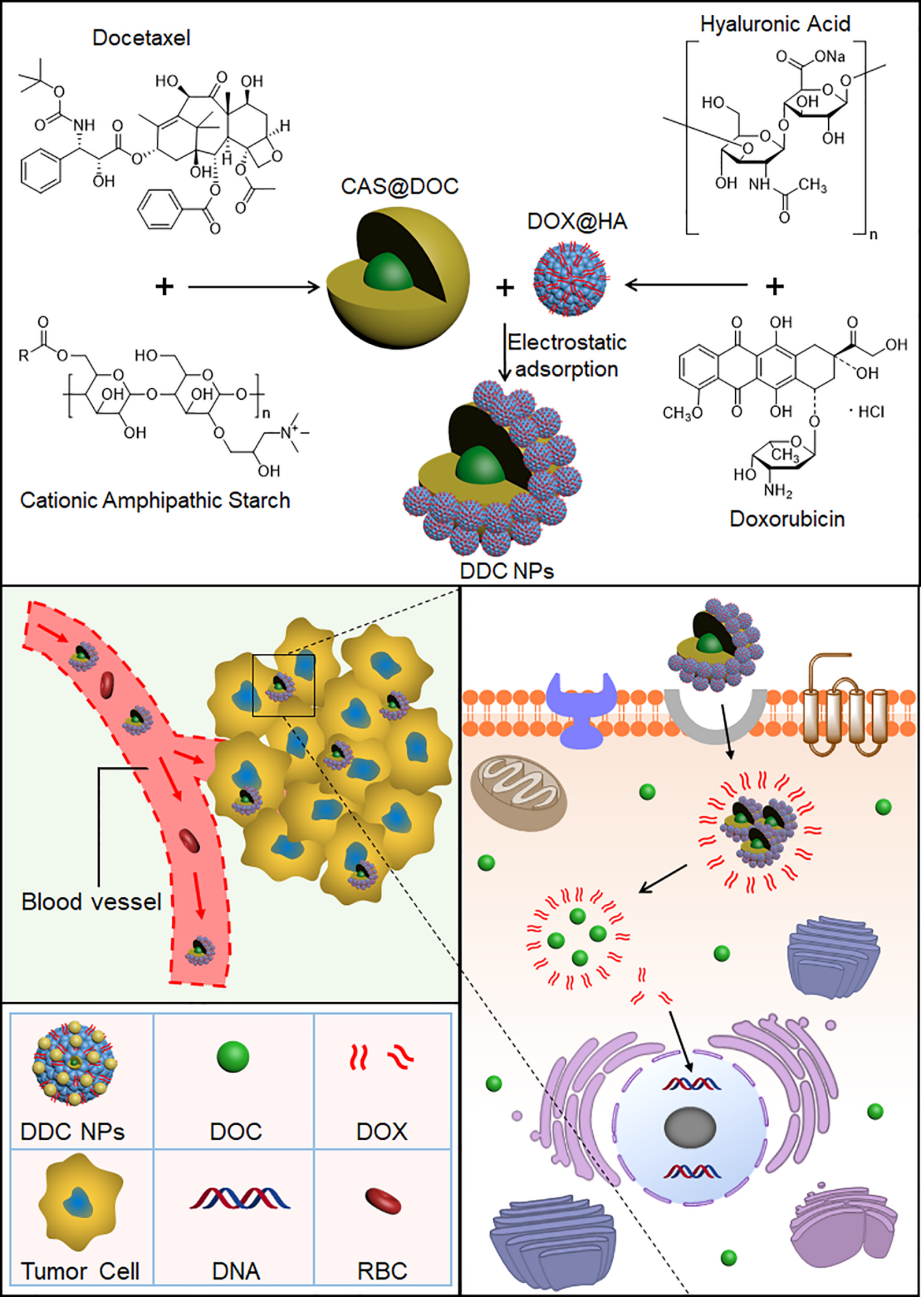
Preparation process of the DDC NPs. DOC and DOX were coencapsulated into the NPs. DDC NPs delivered the drugs into tumor tissues and cells, and then released in cytoplasm.

PCA is a disease that is seriously harmful to male health and impacts quality of life. As the first-line chemotherapeutic medicine in the treatment of PCA, DOC does not have a satisfactory curative effect (Pienta and Smith, 2005). Combination of DOC with other drugs has been developed for the treatment of PCA (**Scheme 1**). Studies have indicated that anthracyclines can enhance the inhibitory effect of DOC against PCA (Pienta, 2001; Kouroussis et al., 2005; Mackler and Pienta, 2005; Neri et al., 2005; Petrioli et al., 2007; Neri et al., 2009). Several studies have verified that the combination of DOC with DOX has a synergistic effect in the treatment of PCA. The following concentrations provided effective synergy in the human PCA cell line PC-3: for DOC, 0.25 to 1 times the IC_50_ value, and for DOX, 2 to 8 times the IC_50_ value (Budman et al., 2002; Tsakalozou et al., 2012). In the present study, the combination of DOC and DOX effectively suppressed all three human PCA cells, which included two that were androgen-independent (PC-3 and DU-145) and one that was Androgen-dependent (Lncap), indicating the broad spectrum of the anti-PCA effect. The optimal proportion of DOX to DOC was 400:1. At the proportions and concentrations used, the combination indexes in the treatments of the three cell lines were below 0.9, indicating that DOC and DOX have excellent synergistic effects against PCA.

To overcome the barriers of pharmacokinetic differences between DOC and DOX, nanotechnology was applied in this study. Nanocarriers are to effectively deliver multiple drugs into tumors while decreasing the accumulation of the drugs in normal organs and tissues, resulting in a reduction in the doses needed and an increased curative effect (Glasgow and Chougule, 2015). The advantages of nano delivery systems benefit combination chemotherapy (Patra et al., 2018). A previous study by our group provided the support, direction, and basis for the current research (Li et al., 2015). Core micelles were used for the encapsulation of apogossypolone. The agent was a hydrophobic compound, as is DOC. Hence, the CSaSt material and the encapsulation vehicle were tested by loading with DOC, and DOC MCs were then prepared. The MCs were spherical, and their size and zeta potential were 45 ± 4.5 nm and 31 ± 2.1 mV, respectively. The properties of the MCs provided an appropriate basis for further NPs construction. The preparation of the DOX NPs and the entire DDC NPs production process were achieved following a previously described process. The primary distinction between the current DDC NPs and previously generated NPs was the drug ratio. In the MLDC NPs, the proportion of DOX and apogossypolone was approximately 1:1 (Li et al., 2015), and in the present study, the ratio of DOC to DOX was 400:1. This difference was significant. Hence, the preparation techniques should be modified to fit current demand. In contrast, the packing materials were provided in excess during the DOC MCs construction step to achieve the objective of having less DOC in the MCs. The amount of DOX NPs was then also increased during the assembly process. At the end of the preparation, the surplus CSaSt and HA were absorbed through electrostatic interactions and removed by centrifugation. The shape of the DDC NPs was a spherical particle with a rough surface, as expected. The size and zeta potential were 68 ± 7.1 nm and -22 ± 2.2 mV, respectively. The changes in size and zeta potential provided further verification of the construction process. The maximum EE values of DOX and DOC were 91.4 ± 3.7% and 96.1 ± 2.3%, respectively. The total DL of the drugs was 9.1 ± 1.7%. The actual ratio of DOX to DOC in the DDC NPs was approximately 350–380:1. At this ratio, the synergy of DOX and DOC was also effective. The release profile of DOX demonstrated that the DDC NPs could gradually release the payload, but when HAase was added, the release was expedited, indicating that the DDC NPs could perform enzymatic release. The stability of the DDC NPs was reliable, and they could be applied *in vitro* and *in vivo*.

The nanocarriers offer a viable choice for use in combination chemotherapy because they can overcome differences in the pharmacokinetics of the drugs and achieve the maximal synergistic effect. Zhang et al. (2007) coencapsulated DOC and DOX in nanocarriers and demonstrated enhanced cytotoxicity against PCA cells. Kolishetti et al. (2010) prepared dual drug nanoparticles for loading DOC and Pt(IV). Their dual drug NPs exhibited 2-fold cytotoxicity in LNCap cells compared with that of the single drug NPs (Kolishetti et al., 2010). The Gu group developed NPs for codelivery of camptothecin and DOX, and the NPs demonstrated excellent inhibition in investigations both *in vitro* and *in vivo* (Tai et al., 2014). An investigation of DDC NP cytotoxicity was initially conducted *in vitro*. The results of the CCK-8 assay demonstrated that the DDC NPs could effectively and synergistically act in both androgen-dependent PCA and androgen-independent PCA cell lines. The clone formation assays further verified that the DDC NPs had obvious cytotoxicity in the PC-3 cells. The DDC NPs exhibited significantly enhanced suppression over either single drug treatment; in addition, there was no obvious difference from the free dual drug treatment. The results further verified that DDC NPs have complete synergy. Moreover, DDC NPs induced effective antimigration in the PC-3 cell line. The effect was not significantly different from the free dual drug-treated group. The effect of the DDC NPs on PCA cell apoptosis was initially assessed by flow cytometry. The apoptosis ratio in the DDC NPs-treated group was much higher than that in the single drug treatments and was similar to that of the dual drug treatment group. The results were further demonstrated by western blotting. The DDC NPs and dual drugs had obviously downregulated Bcl-2 protein, while they obviously induced upregulation of the apoptosis-related factors, such as Bax and cleaved Caspase 3. In general, the DDC NPs with DOC and DOX acted well as curative agents, and the synergistic effect of the two drugs was efficiently realized by the NPs.

The targeted delivery of the DDC NPs was performed by HA. HA is widely used in drug delivery systems because it has ligand-receptor interactions with CD44 (Underhill, 1992). CD44 is expressed at low levels in normal tissues; however, it is pathologically and highly expressed on the surface of tumor cells (Naor et al., 2002; Cichy and Pure, 2003; Naor et al., 2008). Moreover, HA possesses excellent biocompatibility and is biodegradable, nonimmunogenic, and nontoxic, which makes it a favorable alternative to biomaterials, which have raised concerns with respect to these areas (Dosio et al., 2016). The fluorescent label of the NPs has been widely used to observe internalization (Eley et al., 2004; Rivolta et al., 2011). The test of competitively suppressed endocytosis demonstrated that internalization of the DDC NPs was mediated by the ligand-receptor interaction of HA and the CD44 protein. The internalization process of the DDC NPs was directly observed through the distribution of different fluorescence signals. The green fluorescence gradually accumulated in the cytoplasm. The distribution of red fluorescence was clearly distinguished from the green fluorescence, indicated that DOX bound to DNA. The sites where accumulation was observed changed from the cytoplasm to the nucleus. The results indicated that the NPs could effectively deliver the payloads into the cell, where they could be fully released. Thus, the results indicated that DDC NPs could effectively deliver payloads into PCA cells.

The properties of the DDC NPs *in vivo* are critical as application criteria. One important function of NPs is the reduced accumulation of drugs in normal organs and tissues (Byrne et al., 2008). The distribution of the NPs *in vivo* is affected by their size, shape, and surface characteristics (Alexis et al., 2008). NIF imaging and fluorescence labeling *in vivo* were applied to investigate the distribution of DDC NPs *in vivo*. The mouse models were implanted with PC-3 cells. The fluorescent signal in the free IR-780-injected mouse attenuated rapidly, since the majority of the dye was excreted or metabolized. The remaining dye accumulated in the organs and tumors equally. In contrast, the fluorescence signal in DDC NPs-treated mouse exhibited an entirely different mechanism. The initial accumulation sites were the thorax and abdomen, with some found in the lung and liver. It then accumulated in the tumor, where it continuously accumulated. The signal decreased in the abdomen and thorax gradually. This phenomenon was similar to that reported by Yin, in which the fluorescence signals accumulated primarily in the liver and tumor and then attenuated in the liver (Ferguson et al., 2010). The distribution of fluorescence signals in tissues further verified that DDC NPs mainly accumulated in tumors. The results provide strong evidence that DDC NPs possess the ability to target delivery against PCA *in vivo*. Reducing drug accumulation in normal organs and tissues was the primary basis for the decrease in side effects. The results of the acute toxicity test indicated that the DDC NPs could efficiently reduce the toxicity of DOC and DOX *in vivo*. The mortality and pathological changes in DDC NPs-treated mice were significantly lower compared with those in the mice receiving the dual drug treatment. Moreover, the DDC NPs could decrease the occurrence of hemolysis. The hemolysis ratio in the DDC NPs group was obviously lower than that in the free drug-treated group. Antitumor evaluation was the last experiment conducted *in vivo* in the present study. The PC-3 cell xenograft mouse models were used for the experiment. The doses of DOX and DOC were approximately 2 mg/kg and 5 μg/kg, respectively, which were based on the optimal proportion *in vitro*. The results showed that neither the single drug treatment nor the dual drug combination exhibited satisfactory tumor suppression in the mouse models. The DDC NPs effectively suppressed the progression of PCA in the mouse models. The average volume and weight of tumors in the DDC NPs-treated group were significantly smaller than those in the other groups. The histological investigation reflected that tumors in the DDC NPs-treated group exhibited more pathological changes, which indicated that the DDC NPs could deliver more therapeutic agents into tumors. Moreover, the average body weight in the DDC NPs group was significantly greater than that in the other groups. This finding further demonstrated that the antitumor treatment *in vivo* through the NPs was relatively safe.

## Conclusion

In conclusion, the present study demonstrated that the DDC NPs exhibited excellent targeted delivery and inhibition against PCA *in vitro* and *in vivo*. Initially, DOC and DOX at an optimal synergistic ratio were successfully coencapsulated into NPs that were smaller than 100 nm to form DDC NPs. In investigations *in vitro*, the DDC NPs had an equivalent effect when compared with the dual drug combination, including on the parameters for cytotoxicity, antimigration, and induction of apoptosis. The results *in vivo* showed that the synthetically created DDC NPs enhanced the accumulation of drugs in tumor tissues and reduced nonspecific accumulation in normal organs; thus, the NPs effectively enhanced the curative effect while decreasing toxicity *in vivo*. The study suggests that these functional NPs are a platform worthy of development as potential prospects in clinical chemotherapy for PCA.

## Data Availability Statement

The raw data supporting the conclusions of this article will be made available by the authors, without undue reservation, to any qualified researcher.

## Ethics Statement

All animal experiments were conducted following the Guidelines for the Use and Care of Experimental Animals at Xi’an Medical University and were approved by the Laboratory Animal Administration Committee of Xi’an Medical University.

## Author Contributions

KL, RJ, and XC designed the study. KL, WZ, and YC performed the experiments. KL, WZ, YC, RJ, and XC analyzed the results and data. KL, RJ, and WZ prepared the manuscript. RJ and XC modified the manuscript.

## Funding

This study was supported, in part, by the National Natural Science Foundation of China under grant 81801863 and 81660505, the Natural Science Basic Research Program of Shaanxi (2019JQ-485), the Key Research Project of Education Department of the Shaanxi Provincial Government in Shaanxi Province of China (18JS101), the Science and Technology Innovation Base-Open and Sharing Platform of Science and Technology Resources Project of the Shaanxi Province (2019PT-26), the China Nepal Friendship Research Center of Rajiv Kumar Jha Grant (18LJM04), and the Research Foundation of Shaanxi Provincial Research Center for the Project of Prevention and Treatment of Respiratory Diseases in Shaanxi Province of China (2017HXKF04).

## Conflict of Interest

The authors declare that the research was conducted in the absence of any commercial or financial relationships that could be construed as a potential conflict of interest.
